# Neuroendocrine Mechanisms Underlying Non-breeding Aggression: Common Strategies Between Birds and Fish

**DOI:** 10.3389/fncir.2021.716605

**Published:** 2021-07-29

**Authors:** Laura Quintana, Cecilia Jalabert, H. Bobby Fokidis, Kiran K. Soma, Lucia Zubizarreta

**Affiliations:** ^1^Unidad Bases Neurales de la Conducta, Departamento de Neurofisiología Celular y Molecular, Instituto de Investigaciones Biológicas Clemente Estable, Ministerio de Educación y Cultura, Montevideo, Uruguay; ^2^Department of Zoology, The University of British Columbia, Vancouver, BC, Canada; ^3^Department of Biology, Rollins College, Winter Park, FL, United States; ^4^Department of Psychology, The University of British Columbia, Vancouver, BC, Canada; ^5^Laboratorio de Neurofisiología Celular y Sináptica, Departamento de Fisiología, Facultad de Medicina, Universidad de la República, Montevideo, Uruguay

**Keywords:** neurosteroids, territoriality, food intake, testosterone, estradiol, songbird, aromatase, electric fish

## Abstract

Aggression is an adaptive behavior that plays an important role in gaining access to limited resources. Aggression may occur uncoupled from reproduction, thus offering a valuable context to further understand its neural and hormonal regulation. This review focuses on the contributions from song sparrows (*Melospiza melodia*) and the weakly electric banded knifefish (*Gymnotus omarorum*). Together, these models offer clues about the underlying mechanisms of non-breeding aggression, especially the potential roles of neuropeptide Y (NPY) and brain-derived estrogens. The orexigenic NPY is well-conserved between birds and teleost fish, increases in response to low food intake, and influences sex steroid synthesis. In non-breeding *M. melodia*, NPY increases in the social behavior network, and NPY-Y1 receptor expression is upregulated in response to a territorial challenge. In *G. omarorum*, NPY is upregulated in the preoptic area of dominant, but not subordinate, individuals. We hypothesize that NPY may signal a seasonal decrease in food availability and promote non-breeding aggression. In both animal models, non-breeding aggression is estrogen-dependent but gonad-independent. In non-breeding *M. melodia*, neurosteroid synthesis rapidly increases in response to a territorial challenge. In *G. omarorum*, brain aromatase is upregulated in dominant but not subordinate fish. In both species, the dramatic decrease in food availability in the non-breeding season may promote non-breeding aggression, via changes in NPY and/or neurosteroid signaling.

## Introduction

In all vertebrate classes, agonistic behavior is an adaptive social behavior that plays an important role in gaining access to limited resources. Arising early in animal evolution, aggression strongly impacts survival and fitness of individuals, and thus both aggressive behavior and its physiological regulation are under strong evolutionary pressures. This review focuses on two neuroethological models, the song sparrow (*Melospiza melodia*) and the weakly electric banded knifefish (*Gymnotus omarorum)*, and their contributions to understanding the neuroendocrinology of agonistic behavior, particularly territorial aggression.

Although bony fish originated 400 MYA, while birds just 150 MYA, neuroanatomical and functional studies indicate that the neural circuits that regulate social behavior are highly conserved across vertebrates and play similar roles in the regulation of social behaviors (O’Connell and Hofmann, 2012). Originally described in mammals (Newman, 1999), the social behavior network (SBN) consists of reciprocally connected brain regions located in the forebrain, midbrain and hindbrain. More recent work suggests that a broader social decision-making network, which also includes the mesocorticolimbic reward system, regulates adaptive social behaviors in response to different contexts or stimuli. Birds and teleost fish, as well as reptiles and amphibians, all contain this social decision-making network that is homologous with the mammalian counterpart and has similar activation patterns in similar social contexts (O’Connell and Hofmann, 2012). These common features enable comparative studies in different species to establish general principles in the regulation of social behavior, such as aggression, among vertebrates. Both song sparrows and banded knifefish display territorial aggression throughout the year. Although aggression is generally more common in the breeding season, ecological pressures can also lead to territorial aggression in the non-breeding season, a behavior that is displayed in these two species, as well as in mammals (Jasnow et al., 2000, 2002; Trainor et al., 2006). Non-breeding territorial aggression offers a novel context to further understand the underlying mechanisms of aggression.

## Non-Breeding Territorial Behavior

Many animals carefully evaluate the cost–benefit ratios of agonistic interactions since such encounters are very costly in terms of time, energy, and potential injuries. In many species, individuals establish dominant-subordinate relationships to minimize the costs of protracted aggression. The dynamics of aggression are well-studied in both song sparrows and banded knifefish. Both display robust territorial aggression during the non-breeding season.

*Melospiza melodia* is common throughout North America. In the Pacific Northwest, where the climate is humid maritime, song sparrows are sedentary and exhibit year-round territoriality (except briefly during molt) (Wingfield and Hahn, 1994). Aggressive behavior in this species has been widely studied in the field. In a simulated territorial intrusion (STI), a live caged conspecific decoy and song playback are placed in the subject’s territory for 10 or 30 min (Heimovics et al., 2013). During an STI, territorial males exhibit robust and stereotyped aggressive displays that are easily quantifiable. The number of songs, number of flights near the decoy, time spent within 5 m of the decoy, and closest approach to the decoy are recorded as indicators of aggressiveness (Heimovics et al., 2013). Similarly, in a laboratory-STI paradigm, the subject cage is placed adjacent to the decoy cage (with or without conspecific song playback) and the number of barrier contacts and time in proximity to the decoy cage are recorded. In both field and laboratory, males show similar behavioral responses year-round during the STI, although the persistence of aggression after the STI (when the stimuli are removed) is reduced during the non-breeding season (Wingfield, 1994). This reduction of persistence in the non-breeding season is energetically advantageous for these small songbirds (∼25 g body mass) at a time when temperatures are low, days are short, and food is scarce.

*Gymnotus omarorum* inhabits Uruguay, where the climate is humid subtropical. It displays year-round territorial aggression in both males and females, and non-breeding intrasexual aggression is robust and easily quantifiable (Batista et al., 2012; Silva et al., 2013; Quintana et al., 2016). In laboratory settings, the acquisition and defense of territories in non-breeding *G. omarorum* are mediated by agonistic encounters (Perrone et al., 2019). During dyadic encounters in a neutral arena, fish engage in rapid escalating conflicts that resolve in <3 min, with the establishment of a clear dominant/subordinate status. The only known predictor of contest outcome is body size. Agonistic behavior in *G. omarorum* is subdivided into three distinct phases, each with characteristic behaviors. First is a brief evaluation phase that ends with the first attack. Second is a contest phase characterized by overt aggression, where attacks of both contenders correlate positively, showing escalation. Last is a post-resolution phase where the dominant may continue attacking while the subordinate fish retreats without retaliation (Batista et al., 2012; Zubizarreta et al., 2015). In *G. omarorum* contests, subordinates display electric signals in a sequential pattern: first interrupting their electric discharge, then emitting transient electric communication signals in “chirps” and finally, adopting a lower post-resolution discharge rate (Batista et al., 2012; Perrone and Silva, 2018).

Why do animals display territorial aggression in the non-breeding season? It has been proposed that this behavior may arise to secure breeding sites for future reproduction, for shelter, and/or to ensure food resources. In sedentary bird populations in mid to high latitudes, such as song sparrows, territorial aggression in the non-breeding season increases survival by allowing access to food to meet the large energetic costs during cold winters. This seems especially important in hatch-year males, where individuals that gain territories in their first autumn have a higher overwinter survival rate than those that do not (Arcese, 1989). In these latitudes, non-breeding birds face multiple factors impacting metabolism, including reduced food availability, reduced foraging time due to shorter day lengths and inclement weather, and depletion of energy reserves to endure longer overnight fasts during low temperatures (Heimovics et al., 2013). Metabolite profiling reveals non-breeding male song sparrows exhibit lower fat deposition and higher fatty acid oxidation compared to breeding birds (Fokidis et al., 2019). This is consistent with a shift toward a catabolic state with an increased reliance on stored fat reserves, and this could amplify the need for non-breeding aggression to maintain access to a replenishing food supply.

In teleost fish, year-round territoriality also seems to be related to ensuring foraging grounds. Tropical damselfishes establish well-defined year-round territories on corals (Brawley and Adey, 1977; Wallman et al., 2004) where they cultivate algae as a main food source (Lobel, 1980; Sammarco et al., 1983). When fish are not reproductively active, both sexes are highly territorial, fiercely defending their food source (Karino and Kuwamura, 1997; Hata and Umezawa, 2011). *Gymnotus omarorum*, from mid-latitudes, has year-round territoriality that may be due to its need to forage given its extremely high basal metabolic requirements. These animals continuously sense the world around them by producing and receiving electric discharges, a process that is energetically very costly (Markham et al., 2016). Fish that are physically larger also discharge electrical signals of higher amplitude (Caputi and Budelli, 1995) which may contribute to the need for larger foraging grounds. A field study in which the determinants of non-breeding spacing were explored during the winter shows that body size, but not sex, correlates positively with territory size (Zubizarreta et al., 2020a). Oxygen, a limiting physico-chemical variable in aquatic ecosystems, also correlates with territory size. Higher levels of dissolved oxygen may enable fish to defend large territories because their capacity for aerobic respiration is enhanced. The energetic requirements, and thus foraging needs, are probably the same in both sexes during the non-breeding season, and this may explain why territory sizes in the wild are not different between males and females (Zubizarreta et al., 2020a).

## NPY: Mediator of a Seasonal Environmental Cue Promoting Non-Breeding Aggression?

In mid to high latitudes, photoperiod is the most robust environmental factor regulating life cycles. Nevertheless, food availability can be a supplementary cue that allows for year-to-year flexibility (Perfito et al., 2008). Social behavior is intimately linked to feeding at the behavioral level. Moreover, neuropeptides involved in food intake are expressed in the SBN across vertebrates (Fischer and O’Connell, 2017). Among these neuropeptides, the orexigenic neuropeptide Y (NPY), a 36-amino-acid amidated peptide, is particularly important. NPY is extremely well-conserved throughout vertebrate evolution with only a single amino acid differing between mammalian and avian NPY, and 83–85% homology in primary structure between birds and teleost fish (Chartrel et al., 1991; Blomqvist et al., 1992; Larhammar et al., 1992, 1993; Larhammar, 1996). The entire NPY signaling system over the 450 MYA of gnathostome (jawed vertebrate) evolution appears to be under strong stabilizing selection, resulting in structural conservation. Furthermore, other orexigenic neuropeptides, such as orexin, are also well conserved in vertebrates (Zendehdel and Hassanpour, 2014). Investigations into NPY function in bird and fish species have shown that the injection of this peptide stimulates feeding (Kuenzel et al., 1987; Richardson et al., 1995; Strader and Buntin, 2001; Davies and Deviche, 2015; Chen et al., 2016) reviewed in Matsuda et al. (2012) and Volkoff (2019). Fasting, on the other hand, increases NPY gene expression (Boswell et al., 2002; Yang et al., 2018; London and Volkoff,2019a,b), and suppressing NPY decreases food intake (Chen et al., 2016), reviewed in Matsuda et al. (2012). NPY also regulates aggression and/or dominance/subordination in fish (Doyon et al., 2003; Filby et al., 2010; Baran and Streelman, 2020) and mammals (Karl et al., 2004; Emeson and Morabito, 2005; Lischinsky and Lin, 2020). Collectively, these studies demonstrate important physiological and behavioral functions of NPY, thus making it a good candidate for mediating the environmental factors that promote non-breeding territorial aggression in both fish and birds.

In both the song sparrow and banded knifefish, NPY in the SBN might be involved in non-breeding territorial aggression (Mukai et al., 2009; Fokidis et al., 2019; Eastman et al., 2020). In *M. melodia*, NPY immunoreactive cell bodies are found in some regions of the SBN (infundibulum and ventromedial hypothalamus) and the ventral tegmental area (VTA) (Fokidis et al., 2019), whereas fibers are ubiquitous in the SBN. NPY fibers are also present in the nucleus tractus solitarius, which contains specialized neurons that directly respond to changes in extracellular glucose and/or free fatty acids (Mizuno and Oomura, 1984; Blouet and Schwartz, 2012). Thus, NPY might integrate the SBN with metabolic information provided by these glucostatic and lipostatic neurons. Non-breeding song sparrows show elevated NPY in several regions of the SBN, compared to breeding sparrows (Fokidis et al., 2019). Song sparrows challenged with 30 min of STI upregulated gene expression for the NPY-Y1 receptor in the hypothalamus in the non-breeding season, but not in the breeding season (Mukai et al., 2009), suggesting that NPY signaling may respond quickly to changes in the social environment but only during the non-breeding season.

In *Gymnotus omarorum* non-breeding males have NPY transcripts in the POA. Fish dyads that competed over territory and established social hierarchy were subjected to transcriptomic profiling of the POA, and genes related to food intake were robustly clustered according to social phenotype. Dominants, which had acquired the territory through agonistic behavior and displayed exclusive access to its shelter and surrounding area, upregulated NPY and galanin transcripts. Subordinates, which remained in the periphery of the tank avoiding the dominant male, upregulated transcripts of the anorexigenic molecule cholecystokinin (Eastman et al., 2020). This matches a report in other teleost fish which upregulate orexigenic genes (galanin in particular) in dominant fish (Renn et al., 2008). In all, these results support a role for NPY in non-breeding territorial aggression. NPY may be a mediator signaling the seasonal decrease of food availability and promoting the mechanisms specifically underlying aggression in the non-breeding season, a hypothesis that will be tested in the future.

## Neuroestrogens as Key Regulators of Non-Breeding Aggression

Aggressive behaviors that occur outside of the breeding season suggest a role for non-gonadal regulatory mechanisms (reviewed in Jalabert et al., 2018). The independence of non-breeding aggression from gonadal androgens has been well established. In both *M. melodia* and *G. omarorum*, aggression occurs when the gonads are regressed. Furthermore, gonadectomy in the non-breeding season does not affect contest outcome, dynamics, aggression levels, or submissive displays (Wingfield, 1994; Jalabert et al., 2015). Thus, gonadal hormones are not necessary for the expression of aggressive behavior during the non-breeding season in these species. In addition, as in other species that display non-breeding aggression, circulating androgens do not increase in response to territorial challenges (Hau and Beebe, 2011; Vullioud et al., 2013; Ros et al., 2014). However, estrogens have a prominent role in the regulation of non-breeding territorial aggression in both species. Aromatase inhibitors reduce non-breeding aggression in both *M. melodia* and *G. omarorum* (Soma et al., 1999, 2000a,b; Jalabert et al., 2015; Zubizarreta et al., 2020b). Direct actions of androgens, the substrate of aromatase, have been ruled out, as androgen receptor antagonism has no effect on non-breeding aggression (Sperry et al., 2010; Zubizarreta et al., 2020b). The effects of aromatase inhibition are rescued by concurrent estradiol replacement in *M. melodia* (Soma et al., 2000b). In both species, estrogens affect behavior in less than 90 min, which suggests non-genomic actions, most probably produced by locally synthesized steroids.

The brain is an important source of estrogens that promote non-breeding territorial aggression. In *M. melodia*, aromatase mRNA and enzymatic activity are present in the SBN during the non-breeding season (Soma et al., 2003; Wacker et al., 2010). Brain-derived estrogens might be synthesized from precursors such as progesterone or dehydroepiandrosterone (DHEA). Although circulating progesterone levels are similar year-round, progesterone in the SBN is higher in the non-breeding season. This neural progesterone might provide substrate for neural androgen and estrogen synthesis (Jalabert et al., 2021). Circulating levels of DHEA are higher than those of testosterone in the non-breeding season (Soma and Wingfield, 2001), and DHEA can be metabolized in the brain into active androgens and estrogens (Pradhan et al., 2008). In the non-breeding season, a territorial challenge rapidly increases the activity of brain 3β-HSD, an enzyme that converts DHEA to androstenedione (Pradhan et al., 2010). This suggests there is a local increase of aromatizable androgens, which may lead to a rise in local estrogen production. In *G. omarorum*, preliminary results show that estrogens are exclusively brain derived in the non-breeding season in both males and females. Moreover, transcriptomic data from the POA show that aromatase and other steroidogenic enzymes are expressed in the non-breeding season. Males that acquired a stable dominant status after an agonistic encounter show increased brain aromatase transcripts. Conversely, subordinate males show increased expression of transcripts involved in the conversion of androgens away from estrogens and toward non-aromatizable androgens (Eastman et al., 2020).

## A Hypothesis on the Regulation of Non-Breeding Aggression

**FIGURE 1:**
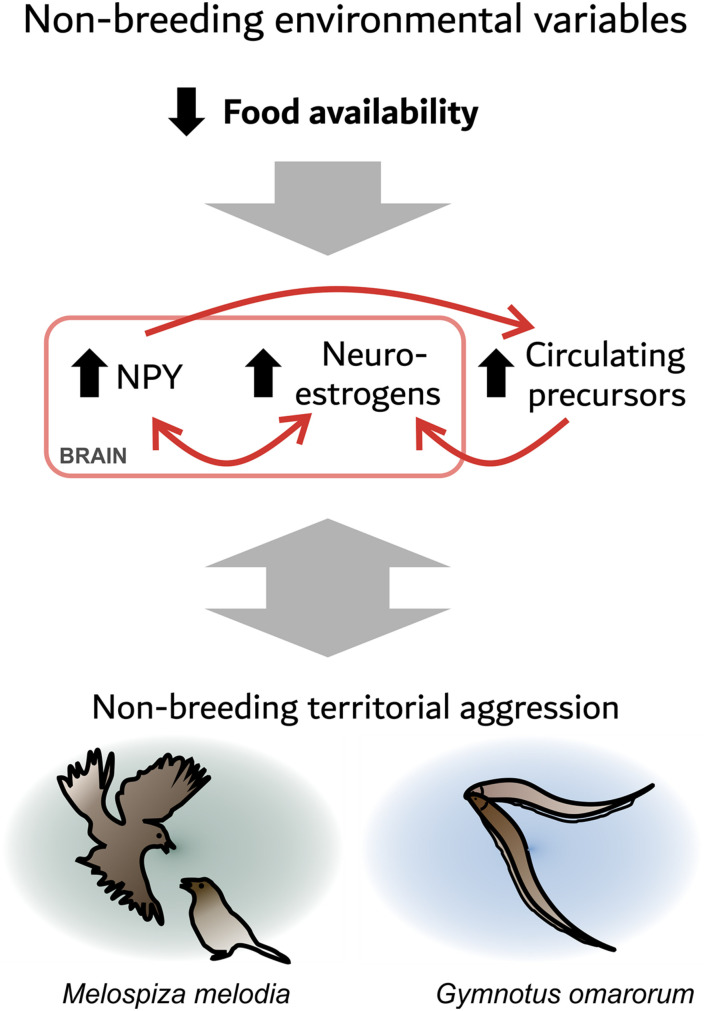
Food availability may be an environmental factor that modulates the expression of territorial aggression through neuropeptide Y (NPY) and neurosteroid signaling. A seasonal decrease in food availability increases NPY in the social behavior network (SBN). NPY may stimulate non-breeding territorial aggression directly, or via the production of neuroestrogens. In addition, a decrease in food availability may increase circulating precursors to neuroestrogens. Agonistic encounters also affect the neuroendocrine state as dominants show an increase in NPY and aromatase expression, which may reinforce the defense of the foraging territory.

Many studies link food intake physiology and sex steroids. For example, in the gymnotiform *Brachyhypomus gauderio*, long-term food restriction increases circulating androgens, as well as electric signaling in response to social challenge (Gavassa and Stoddard, 2012). In the zebra finch (*Taeniopygia guttata*), an acute fast decreases plasma testosterone levels, but increases plasma DHEA levels and estrogen levels in the VTA and periaqueductal gray (Fokidis et al., 2013). These areas contain aromatase (Shen et al., 1995) and NPY (Fokidis et al., 2019). Furthermore, fasting increases agonistic behavior in this otherwise gregarious species (Fokidis et al., 2013). In fish, NPY is present in key neuroendocrine regulatory centers, such as the POA, and is regulated by sex steroids (Peng et al., 1994). In turn, NPY has seasonal actions on gonadal sex steroid production through its stimulation of pituitary gonadotrophins (Kah et al., 1989; Kalra and Crowley, 1992; Peng et al., 1994; Yaron et al., 2003). Collectively, these data suggest an evolutionary conserved relationship between food intake and sex steroids that is mediated at least partly by NPY signaling. These observations suggest the hypothesis that decreased food availability during winter increases brain NPY signaling, which stimulates neuroestrogen synthesis and thus aggression. NPY might also affect aggression *via* other mechanisms, such as serotonin neurotransmission (Karl et al., 2004; Figure 1). In addition, agonistic encounters affect NPY and neurosteroid signaling, reinforcing the defense of the foraging territory. The similarities between the two species highlighted here might be relevant for understanding non-breeding territorial aggression in other species.

## Author Contributions

All authors listed have made a substantial, direct and intellectual contribution to the work, and approved it for publication.

## Conflict of Interest

The authors declare that the research was conducted in the absence of any commercial or financial relationships that could be construed as a potential conflict of interest. The reviewer JW declared a past co-authorship with the authors CJ and KS to the handling editor.

## Publisher’s Note

All claims expressed in this article are solely those of the authors and do not necessarily represent those of their affiliated organizations, or those of the publisher, the editors and the reviewers. Any product that may be evaluated in this article, or claim that may be made by its manufacturer, is not guaranteed or endorsed by the publisher.
